# Management of Vulvovaginal Cellular Angiofibroma: A Single-Center Experience

**DOI:** 10.3389/fsurg.2022.899329

**Published:** 2022-05-27

**Authors:** Zhen Yuan, Jinhui Wang, Yongxue Wang, Fengzhi Feng, Lingya Pan, Yang Xiang, Xiaohua Shi

**Affiliations:** ^1^ Department of Obstetrics and Gynecology, Peking Union Medical College Hospital, Chinese Academy of Medical Sciences & Peking Union Medical College. National Clinical Research Center for Obstetric & Gynecologic Diseases, Beijing, China; ^2^ Department of Pathology, Peking Union Medical College Hospital, Chinese Academy of Medical Science & Peking Union Medical College, Beijing, China

**Keywords:** cellular angiofibroma, surgical treatment, medical treatment, relapse, gynecologic surgery

## Abstract

**Objective:**

The study aimed to explore the clinical characteristics, treatment, and prognosis of cellular angiofibroma in females.

**Methods:**

We performed a retrospective study in patients with vulvovaginal cellular angiofibroma treated at Peking Union Medical College Hospital between August 2012 and October 2021.

**Results:**

Eight patients were included in our study, with 7 cases of vulvar tumors and 1 case of vaginal stump tumors. The median age at diagnosis was 47.5 years (range, 38–83 years). The tumors were found incidentally in two patients (2/8, 25.00%) without specific history before diagnosis surgery. Of the other six patients, the median history from onset of the mass to diagnosis was 5.5 years (range, 3–14 years). Complete excision was performed in all 8 patients. According to histopathologic examination, the median tumor size was 3.4 cm (range, 1.7–11 cm). As the tumor size increased, both the operation time and postoperative length of stay increased. Gonadotrophin releasing hormone agonist was used in one case to minimize the size of the tumor, obtaining satisfactory results. Up to the last follow-up, no evidence of relapse was found in all 8 patients.

**Conclusions:**

For vulvovaginal cellular angiofibroma, the mainstay of treatment remains surgical resection without residual tumor if possible; inadvertent urinary system injury and rectum injury should be avoided to the utmost; and enough attention should be paid to hemostasis to avoid hematoma after surgery. Before surgery, hormone receptor modulators may be considered to minimize the size of the tumor to reduce the surgery-associated risk.

## Introduction

First described by Nucci et al. in 1997, the name “cellular angiofibroma” emphasized the two principal components of this tumor: the cellular spindle cell and the prominent blood vessels (1). Cellular angiofibroma predominantly occurs in the superficial soft tissues of the vulva in the female or inguinoscrotal region in the male, respectively (2). For soft tissue angiofibroma, the main treatment has been surgical resection with negative margins (3, 4). Considering the unique location of female cellular angiofibroma, the aim of this study was to explore the clinical characteristics, treatment, and prognosis of this tumor.

## Materials and Methods

We retrospectively analyzed patients with vulvovaginal cellular angiofibroma at Peking Union Medical College Hospital (PUMCH) between August 2012 and October 2021. Patients’ information was collected from their medical records, including the age of onset, clinical features, treatment, and outcome. At least two pathologists from the Department of Pathology at the PUMCH confirmed the diagnosis of cellular angiofibroma. The follow-up information was obtained from outpatient medical records and telephone interviews.

This retrospective study was approved by the PUMCH ethical committee and the requirement for written informed consent was waived. The dataset was de-identified to protect patients’ privacy.

## Results

### Clinical Characteristics

A total of eight female patients were diagnosed with cellular angiofibroma in our hospital. There were seven cases of vulvar tumors and one case of vaginal stump tumor. The clinical characteristics of the included patients are summarized in Table 1.

**Table 1:** The clinical characteristics of the included patients.

Case	Age	Gravidity and parity	BMI	Chief complaint	Time since the onset of the mass	Pre-operation imaging evaluation	Pre-operation diagnosis	Surgery	Follow-up
1	38	G2P2	23.11	Vulvar neoplasm	5 years	BUS: tumor of 4.1 × 2.5 × 1.2 cm, with blood flow signal	Vulvar neoplasm	Vulvar tumor resection	DFS, 88 m
2	45	G1P1	23.51	Vulvar neoplasm	10 years	N/A	Bartholin gland cyst	Bartholin gland cyst resection and vaginal tumor resection	DFS, 12 m
3	50	G1P1	24.95	Vulvar neoplasm	14 years	BUS: tumor of 5.4 × 2.4 × 1.3 cm, with blood flow signal	Vulvar neoplasm	Vulvar tumor resection	DFS, 2 m
4	59	G1P1	24.22	Vulvar neoplasm	3 years	BUS: tumor of 5.3 × 2.3 × 1.3 cm, with blood flow signal	Vulvar neoplasm	Vulvar tumor resection	DFS, 2 m
5	83	G2P2	28.30	incidentally found	Unknown	N/A	Vaginal stump polyp	Vaginal stump polyp resection	DFS, 15 m
6	38	G1P1	23.71	Vulvar aggressive angiomyxoma relapsed 2 years after resection and continued to grow for 6 years	6 years	CT: tumor of 14.6 × 7.2 × 5.6 cm, and closely attached to the uterus, cervix and vagina	Vulvar aggressive angiomyxoma relapse	Laparoscopic exploration and vulvar tumor resection	DFS, 112 m
7	42	G0P0	25.25	AUB, vulvar neoplasm	4 years	N/A	Vulvar neoplasm	Vulvar tumor resection	DFS, 97 m
8	64	G5P3	34.77	PMB, incidentally found	Unknown	N/A	Vulvar neoplasm	Vulvar tumor resection	DFS, 85 m

*BMI, body mass index; DFS, disease-free survival; BUS, Type B ultrasonic inspection; CT, computerized tomography; AUB, abnormal uterine bleeding; PMB, postmenopausal bleeding.*

The median age at diagnosis was 47.50 years (range, 38–83 years). The median body mass index was 24.58 kg/m^2^ (range, 23.11–34.77 kg/m^2^). Four patients (4/8, 50.00%) were in menopause. One patient (1/8, 12.50%) was nulliparous.

The tumors were found incidentally in two patients (2/8, 25.00%) without specific history before the diagnostic surgery. In the other six patients, the median history from onset of the mass to surgery was 5.50 years (range, 3–14 years). At the beginning of the history, there was no symptom presented. Regarding associated symptoms before diagnosis surgery, six patients felt continuous enlargement of mass (case 1, 2, 3, 4, 6 and 7), one patient had distending pain (case 2), and two patients reported uncomfortable during sitting and ambulating (case 1 and 6).

Regarding previous surgical history, one patient underwent total hysterectomy and bilateral salpingo-oophorectomy due to uterine myoma (case 5), and one patient underwent resection of an aggressive angiomyxoma occurring in the deep soft tissue of the vulvovaginal region and pelvis (case 6).

### Preoperative Evaluation

Clinicians performed physical examinations for all eight patients before surgery. The masses arose in the vulvar region in seven patients and the vaginal stump in one patient. Patients whose masses were in the vulvar region were without skin lesion by visual inspection and painless by palpation. The mass arising in the vaginal stump was like a polypus.

Four patients underwent preoperative imaging evaluation, and blood flow signals were found in three of the patients who underwent ultrasound. By the imaging evaluation, the tumors ranged from 4.1 to 14.6 cm, with a median size of 5.4 cm. Among the other four patients without imaging evaluation, the tumors ranged from 1.5 to 3.0 cm with a median 2.0 cm. Interestingly, for case 2, a physical examination under anesthesia revealed two masses, one underlying the left Bartholin gland and one in the left vulvovaginal region reaching deep soft tissue, respectively.

Among the eight patients, one case of Bartholin gland cyst, one case of relapse of aggressive vulvar angiomyxoma, one case of vaginal stump polypus, and five cases of vulvar neoplasm were diagnosed preoperatively. The reasons for tumor resection varied among the eight patients. Cases 1 and 6 were due to discomfort when sitting and ambulating, case 2 was misdiagnosed as Bartholin gland cyst with distending pain, case 3 was suspected to be malignant by imaging evaluation, case 4 was continuous enlargement, and case 5 was mistaken for vaginal stump large polyps. For cases 7 and 8, abnormal uterine bleeding and postmenopausal bleeding triggered them for surgery, respectively, and vulvar tumor masses were resected simultaneously.

### Surgery

All eight patients had complete resection of their tumors. With the exception of case 5, the remaining seven patients were operated in an operating room under general anesthesia. These seven patients were catheterized during surgery to avoid anterior bladder injury. Rectal palpation was performed in cases 2 and 6 to prevent posterior anorectal injury. Depending on the tissue involved, interrupted or figure-of-eight stitches and by one- or multi-layered stitches were used to close the space created by the tumor excision to avoid hematoma formation. In this study, none of these eight patients underwent reconstructive surgery which need one or more flap types. Except case 5 in which the cut needn’t to be stitched, the remaining seven patients underwent primary closure of the wound edges without reconstructive surgery. Additionally, in case 2, a sandbag was placed in the vagina to maintain pressure since the cellular angiofibroma was closely proximal to the vaginal wall. Except for case 6, all seven patients recovered without surgery-associated complications.

**Figure 1:**
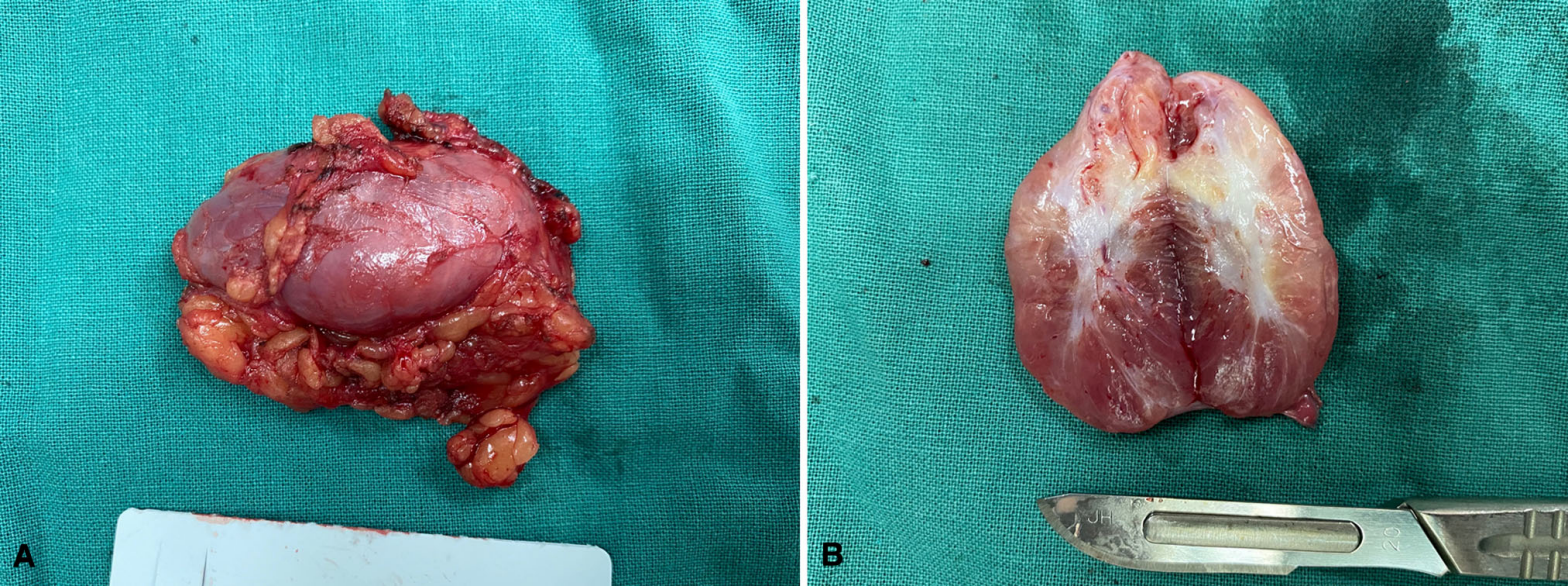
The macroscopic appearance and cut surface. (**A**) The surface of the tumor, (**B**)The cut surface of the tumor.

**Figure 2:**
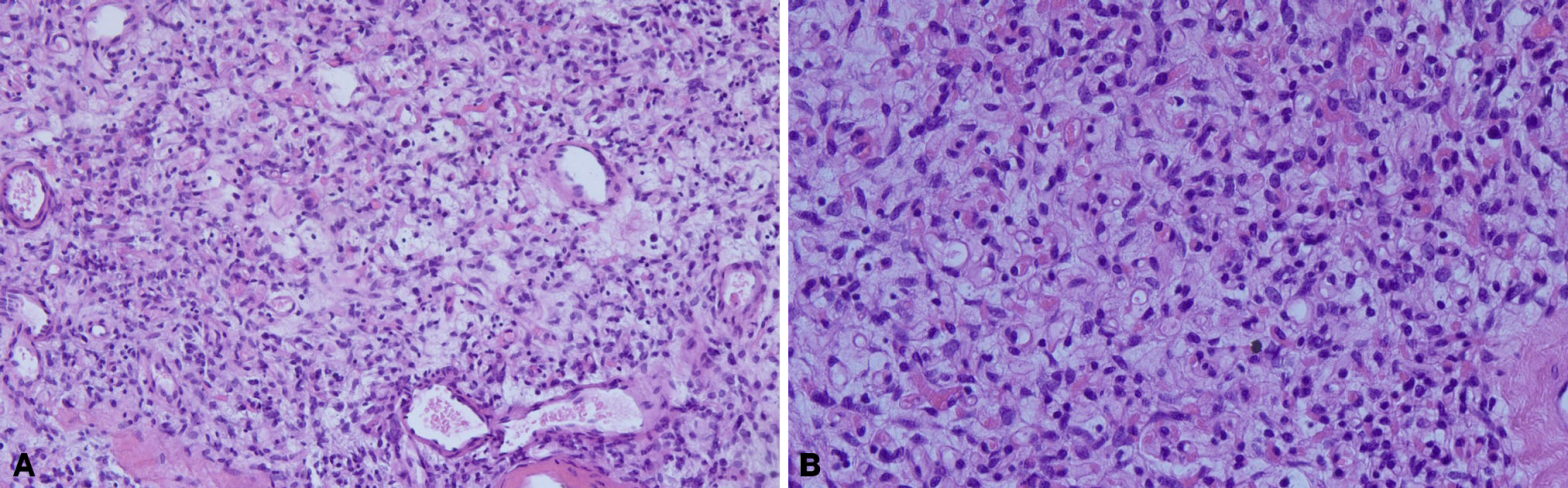
The histopathological examination. Spindle-shaped cells resting in the background of fibrous stroma containing delicate collagen fibers and numerous small to medium-sized thick-walled blood vessels. (**A**) ×100, (**B**) ×200.

**Figure 3:**
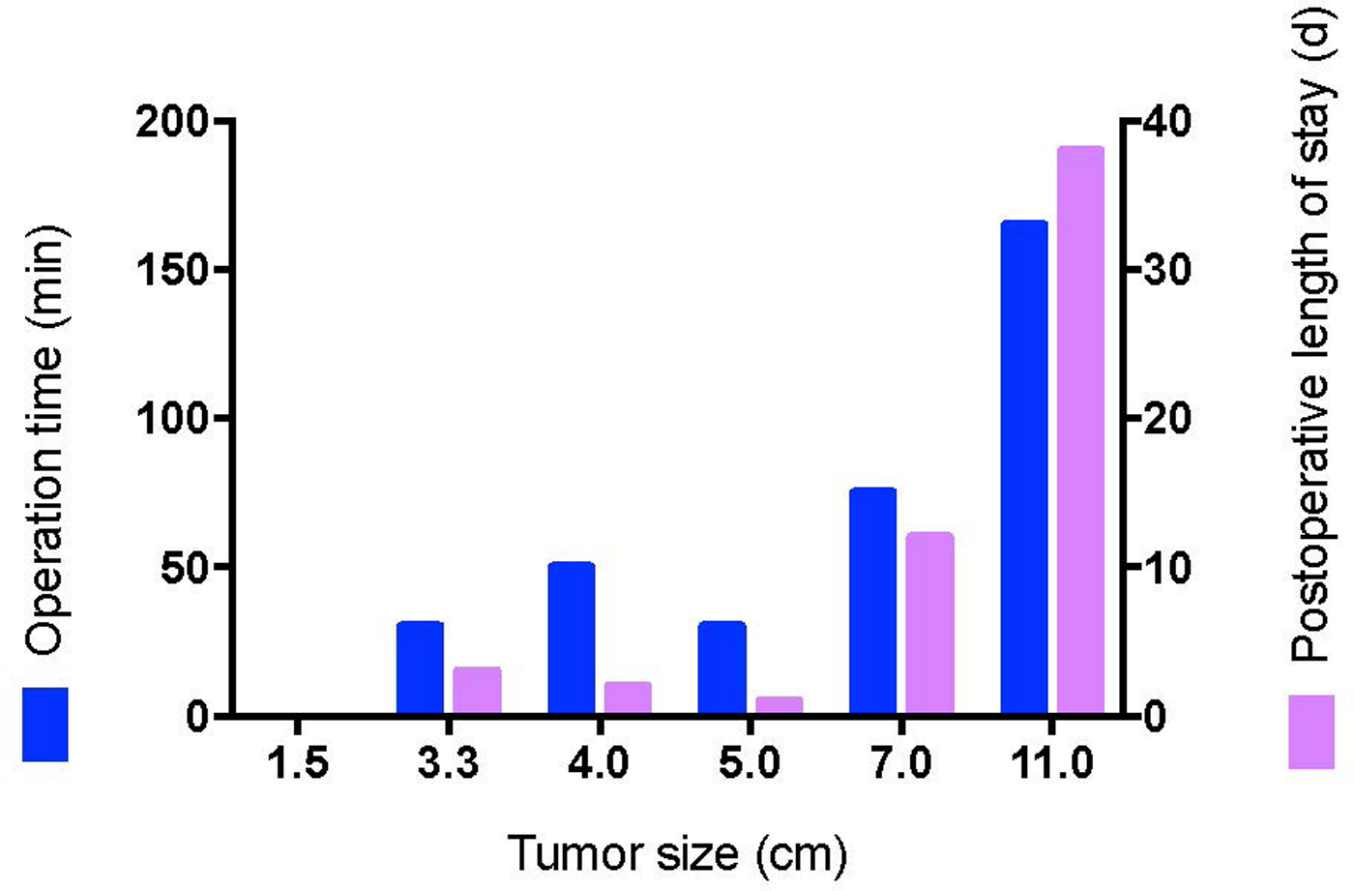
The relationship between tumor size and perioperative events.

Except for cases 7 and 8 who underwent other surgeries at the same time, the operation time ranged from 0 to 165 min (median time: 40.0 min), and the postoperative length of stay ranged from 0 to 38 days (median day: 2.5 days). Histopathologic examination revealed tumors ranging from 1.7 to 11 cm (median size: 3.4 cm). The macroscopic appearance and cut surface are shown in Figure 1 (case 3), and histopathological examination is shown in Figure 2 (case 3). Except for cases 7 and 8, with the tumor size increasing, both the operation time and postoperative length of stay increased with increasing tumor size, as displayed in Figure 3. No evidence of recurrence was found in all eight patients until the last follow-up visit.

### Medical Treatment

Interestingly, one patient (case 6) received preoperative medical treatment with satisfactory results. Case 6 had a history of aggressive vaginal angiomyxoma resection at another hospital. The tumor relapsed 2 years later by clinical evaluation and kept growing to 16.4 × 10.6 × 10.2 cm on ultrasound during the subsequent 3 years. Gonadotrophin releasing hormone agonist (GnRH-a) was given for 3 years, and the tumor was once reduced to 7.5 × 5.8 × 5.4 cm on ultrasound. Five months before the surgery, a CT examination showed a tumor of approximately 14.6 × 7.2 × 5.6 cm and closely attached to the uterus, cervix, and right lower side of the vagina. GnRH-a was given continuously for 5 months before the second surgery, and laparoscopic exploration of the abdominopelvic cavity revealed no tumor. The vulvar tumor was subsequently resected. The tumor had poorly defined borders with surrounding normal tissue and was adjacent to the vagina and anorectum, and the abundant vascular structure supported the vulvovaginal region. The surgery was difficult and was completed after inserting a drainage tube into the surgical area. The tumor size was 11 × 8 × 5 cm, with a slightly rough surface in parts on pathological examination. The cut surface was greyish-pink with a rubbery and delicate feel in some areas. Vascular-like cavities were seen in some areas with a 0.1–0.2 cm diameter. Immunohistochemical analysis showed positive staining for the cluster of differentiation (CD34), estrogen receptor (ER), and partial progestogen receptor (PR). In addition, Desmin and protein S-100 were negative. Postoperatively, an intestinal-vulvar fistula occurred, which healed well after conservative treatment.

## Discussions

Cellular angiofibroma usually arise in the superficial soft tissues of the female vulva (2). In 2020, cervicovaginal cellular angiofibroma, a large mass bulging in the posterior fornix, was first published (5). Our study reported vaginal stump cellular angiofibroma for the first time. Therefore, cellular angiofibroma should also be considered in the differential diagnosis of a painless polypus vaginal stuff mass.

Our study found blood flow signals in all three patients evaluated by ultrasound imaging. In a previous study, magnetic resonance imaging evaluation demonstrated the feature of prominent vascularity (6). It is an interesting feature consistent with the pathologic characteristics of a vascular-rich fibroblastic neoplasm (7).

In our study, the treatment of cellular angiofibroma included both medical treatment and surgical treatment. Surgical treatment is the cornerstone of treatment for cellular angiofibroma (2, 8). Regarding surgery, we believe several issues should be considered.

First, we believe the tumors should be treated by simple residue-free excision if possible. In previous studies, extensive resection was not required, considering even positive surgical margins, including one case of incomplete tumor resection with no reported recurrences or metastasis (2, 9). However, in our study, for case 6, who had a history of “aggressive vaginal angiomyxoma” resection at another institution, the tumor relapsed 2 years later, and the recurrent tumor was confirmed to be cellular angiofibroma in our hospital. It was impossible to reconfirm whether the prior “aggressive vaginal angiomyxoma” was indeed cellular angiofibroma and whether the previous “aggressive vaginal angiomyxoma” was completely or incompletely excised. However, in our opinion, the previous “aggressive angiomyxoma” was most likely cellular angiofibroma and the previous resection was incomplete. Consistent with previous studies, in our study, patients with cellular angiofibroma presented with slow-growing and painless masses (2, 7), and the median history of cellular angiofibroma was 5.5 years, without associated symptoms at the beginning of the history. Considering above-mentioned factors, the recurrence could be identified with enough follow-up time. Consistent with our reviews, in an analysis of cellular angiofibroma in the male, eleven men were followed for 1–13 years, four of whom were lost to follow-up, and one of whom developed a local recurrence (10). Considering the relatively high risk of surgery-associated complications of reoperation, we believe that tumors should be treated by simple tumor excision without residue, if possible. Additionally, regarding wether reconstructive surgery should be performed. Generally, for vulvar caner, surgeons always aim for surgical margins of 2 cm to achieve pathological margins of at least 8 mm to minimize local disease recurrence, removing a large amount of tissue (11).In these circumstances, reconstructive surgery is often necessary, after radical wide local excision of tumor or radical vulvectomy, to reduce the risk of postoperative complication (12–15). Female cellular angiofibroma predominantly occurs in the superficial soft tissues of the vulva (2). And the surgery of vulvovaginal cellular angiofibroma in our study was simple residue-free tumor excision without exorbitant tension of the skin closure and distortion of the anatomy. Therefore, in our study, primary closure of wound was accompanied without reconstructive surgery. Consistent with our opinion, after tumor excision, primary closure rather than reconstructive surgery was performed in a previous retrospective study (12). However, we are in favor of the idealism of reconstructive surgery, to obtain tension free skin closure with good quality tissues, to fill dead space, to restore normal anatomy, and to maintain function (16). For case 6, in which an intestinal-vulvar fistula occurred, reconstructive surgery may stand a chance of avoiding this complication. And the surgery should continue to improve in the future.

Second, accidental urological and rectum injuries should be avoided to the utmost. In our study, catheterization was used and no urethral injury was found in all cases. Cellular angiofibroma arose most commonly in the superficial soft tissue and was well-defined, but in some cases, the tumors are located in deep soft tissues and grow infiltratively (17). In our study, in cases 2 and 6, the tumors reached deep soft tissues. And rectal palpation was performed to avoid injury to the posterior rectum in cases 2 and 6. Even with rectal palpation, for case 6, unfortunately, an intestinal-vulvar fistula occurred postoperatively. It seems that the secondary surgery and the relatively large size of tumor reaching deep soft tissue may be two critical factors contributing to this adverse event. Moreover, as was shown in Figure 3, both the operation time and postoperative length of stay increased with the increase in tumor size. Therefore, a combination of the factors mentioned above should be considered when deciding the time of surgery.

Third, intraoperative and postoperative attention should be paid to hemostasis to avoid hematoma. Since the vulva mostly consists of loose connective tissue and smooth muscle with a rich vascular structure, damage to the labial branches of the internal pudendal artery within this vascular network can quickly form a hematoma (18). In our study, interrupted or figure-of-eight stitches, one- or multi-layered stitches, insertion of a drainage tube into the surgical cavity, and insertion of a sandbag in the vagina to maintain pressure were used to avoid the formation of pockets hematomas. Consistent with our study, the vulvar hematoma was treated by inserting a drain into the hematoma cavity for at least 24 h, inserting a tampon into the vagina, and inserting a foley catheter were once proposed in the previous study (19).

Finally, there are no previous reported studies on the medical treatment of cellular angiofibroma. In our study, for case 6, GnRH-a was used for 3 years, and the tumor size once was minimized from 16.4 × 10.6 × 10.2 cm to 7.5 × 5.8 × 5.4 cm. Immunohistochemical analysis of resected cellular angiofibroma showed positive staining for ER and partial staining for PR. In a previous study, ER and PR were expressed in 50–90% female cellular angiofibroma (8). Even male cellular angiofibroma in the anorectal region expressed ER and PR variably (9). To our knowledge, there are no previous studies regarding the antiproliferative effects of receptor modulators in cellular angiofibroma. Similarly, Schick et al. have reported the expression of sex hormone receptors in juvenile angiofibroma and the antiproliferative effect of receptor modulators (20). Considering the increased operation time and postoperative length of stay with increasing tumor size, hormone receptor modulators, such as GnRH-a, could be considered before surgery to minimize tumor size in order to reduce the surgery-associated risk. Similarly, in other types of tumors, hormonal therapy is also a valuable field of investigation (21).

This study was limited by the inadequate large sample size and its retrospective nature, which may introduce a degree of bias. Despite these limitations, several interesting findings were observed in our study. The first one is our first report of vaginal stump cellular angiofibroma. The second is that we reported a suspicious recurrence of cellular angiofibroma in the female. Finally, the third important finding concerns the antiproliferative effects of hormone receptor modulators in cellular angiofibroma.

In conclusion, for cellular angiofibroma in the female, the mainstay of treatment remains surgical resection without residual tumor if possible; accidental urinary and rectum injury should be avoided to the utmost; and intraoperative and postoperative attention should be paid to hemostasis to prevent hematoma. Before surgery, hormone receptor modulators may be considered to minimize the tumor size to reduce the surgery-associated risk.

## Data Availability

The original contributions presented in the study are included in the article/Supplementary Material, further inquiries can be directed to the corresponding author/s.
